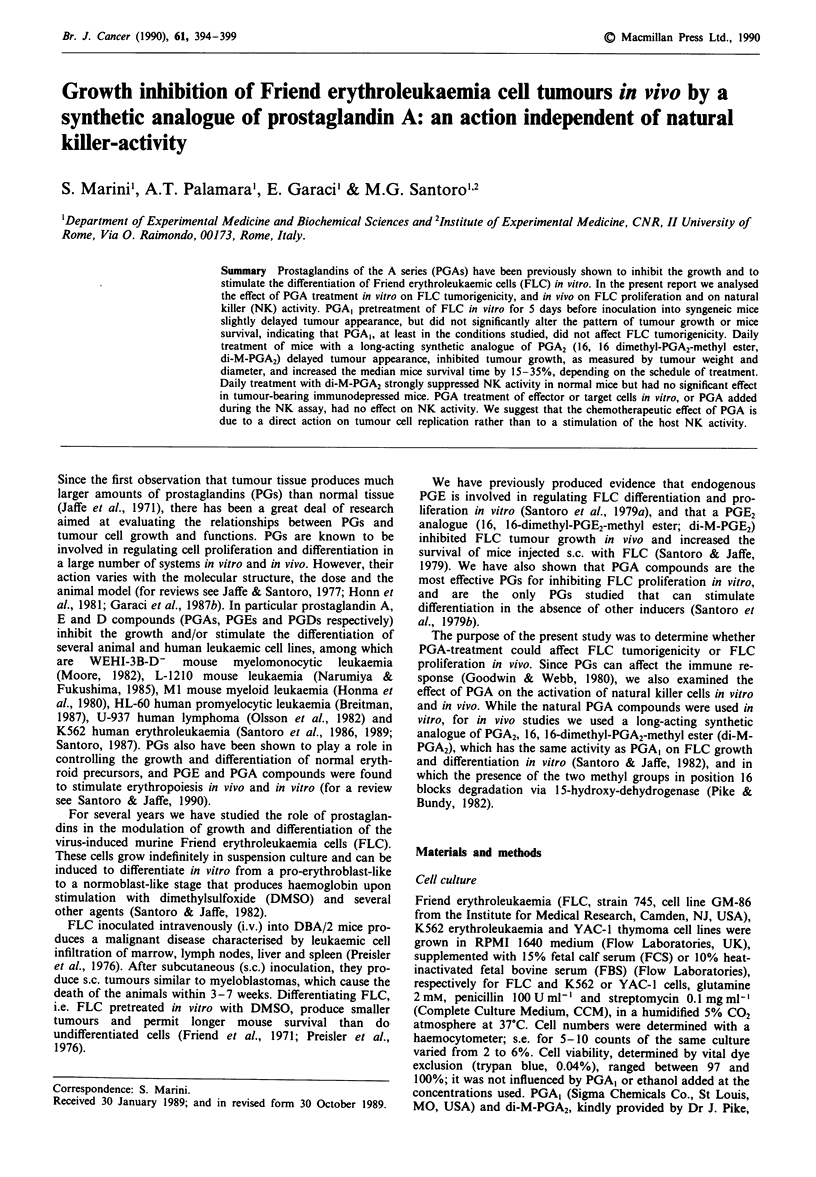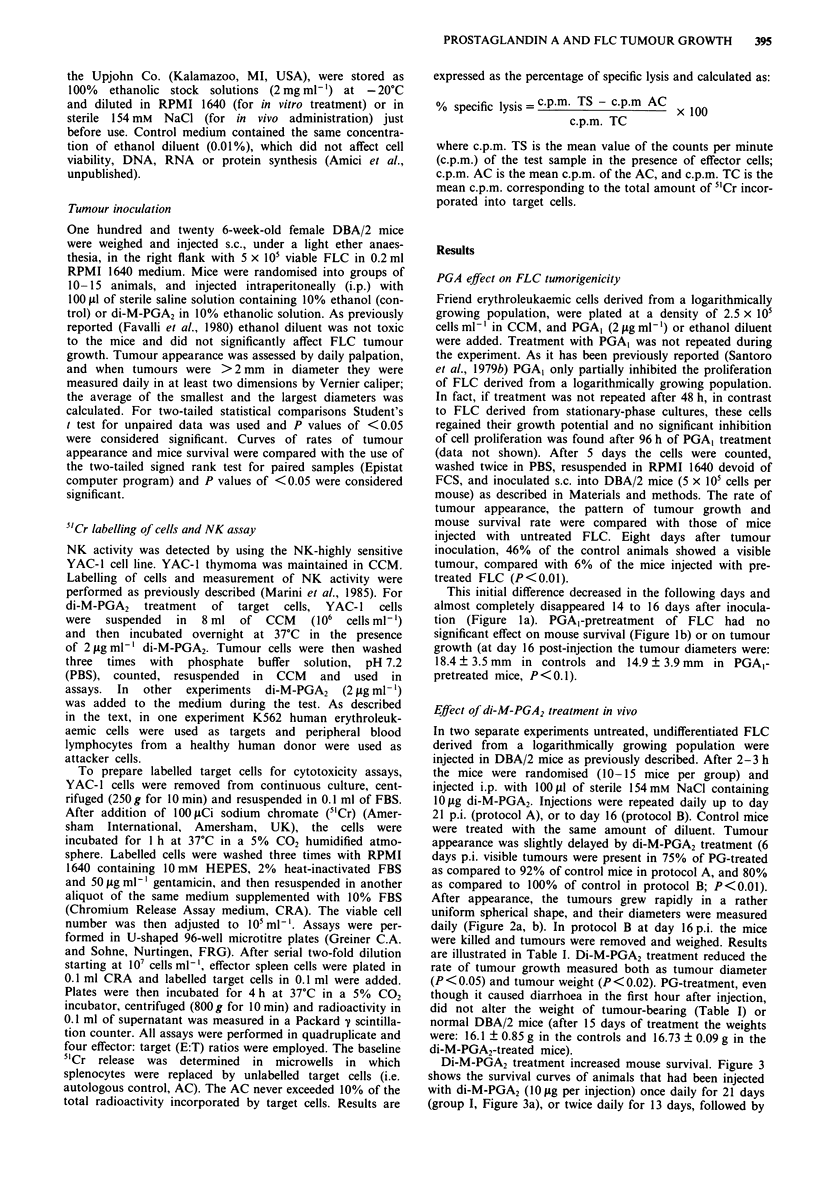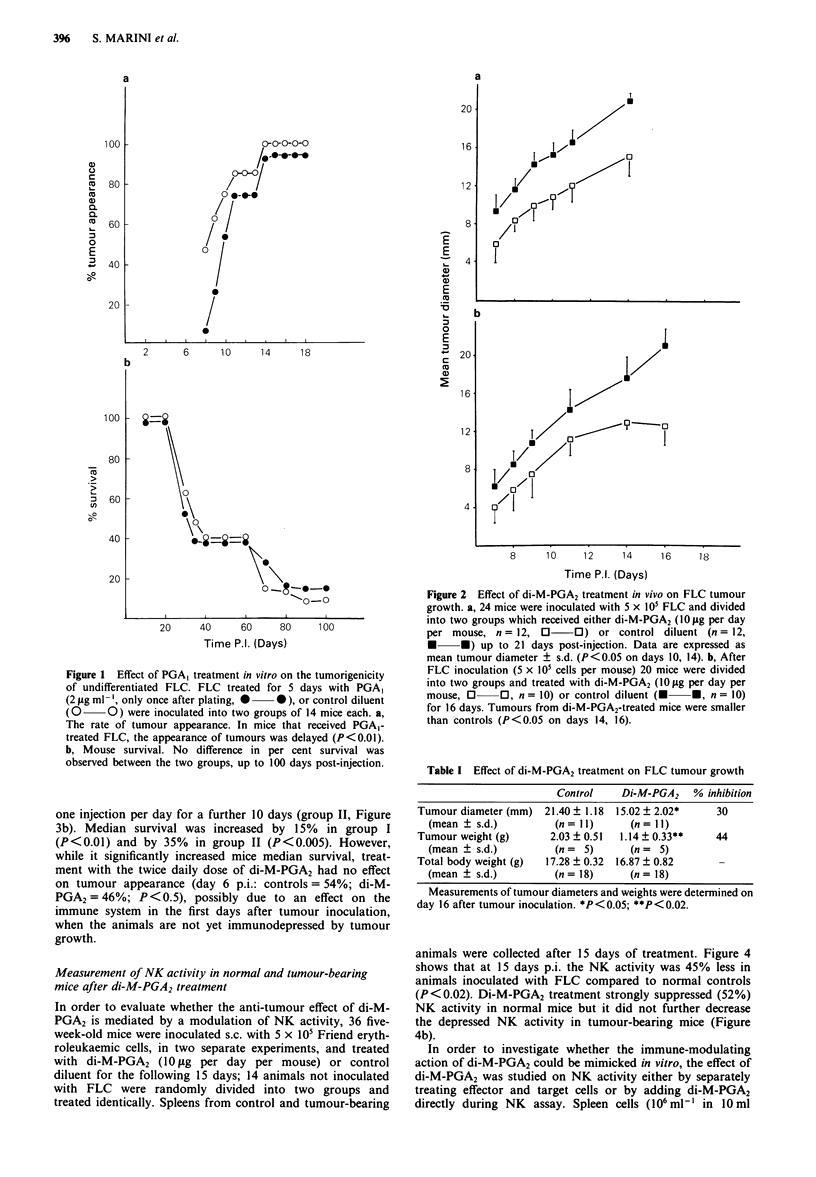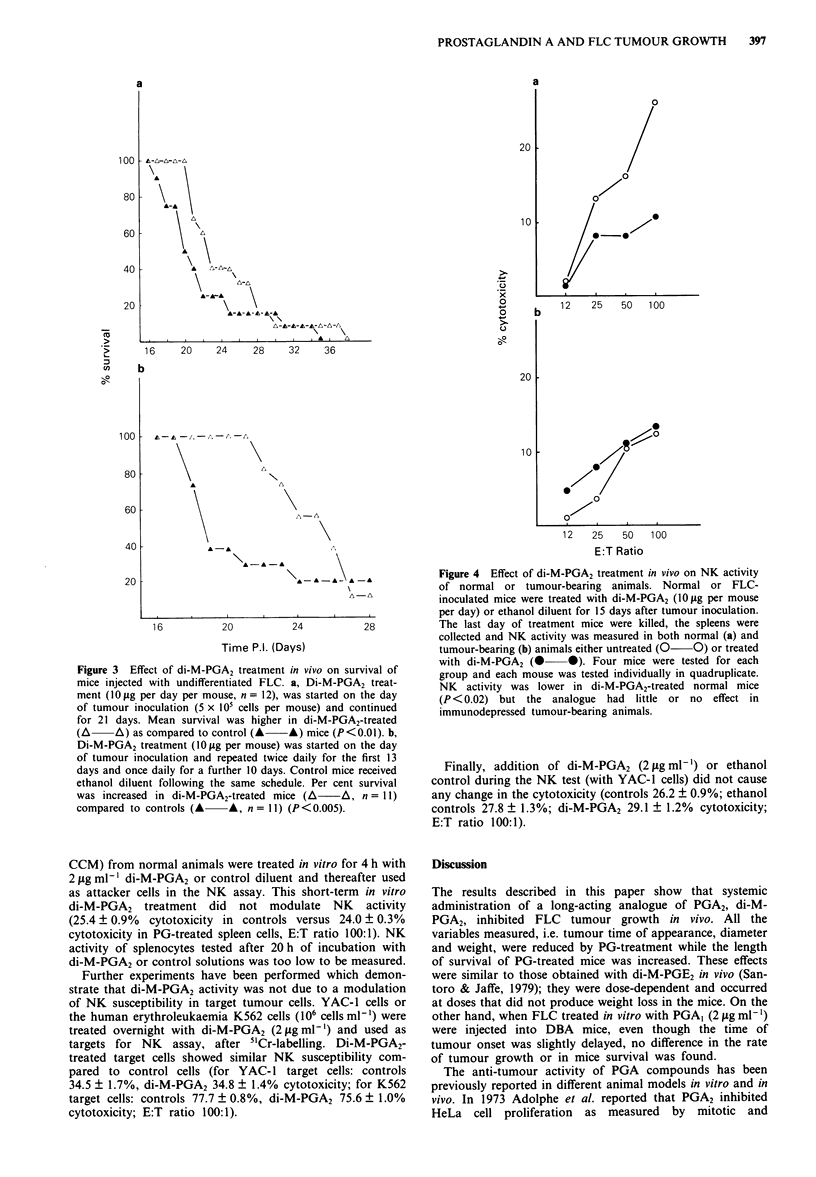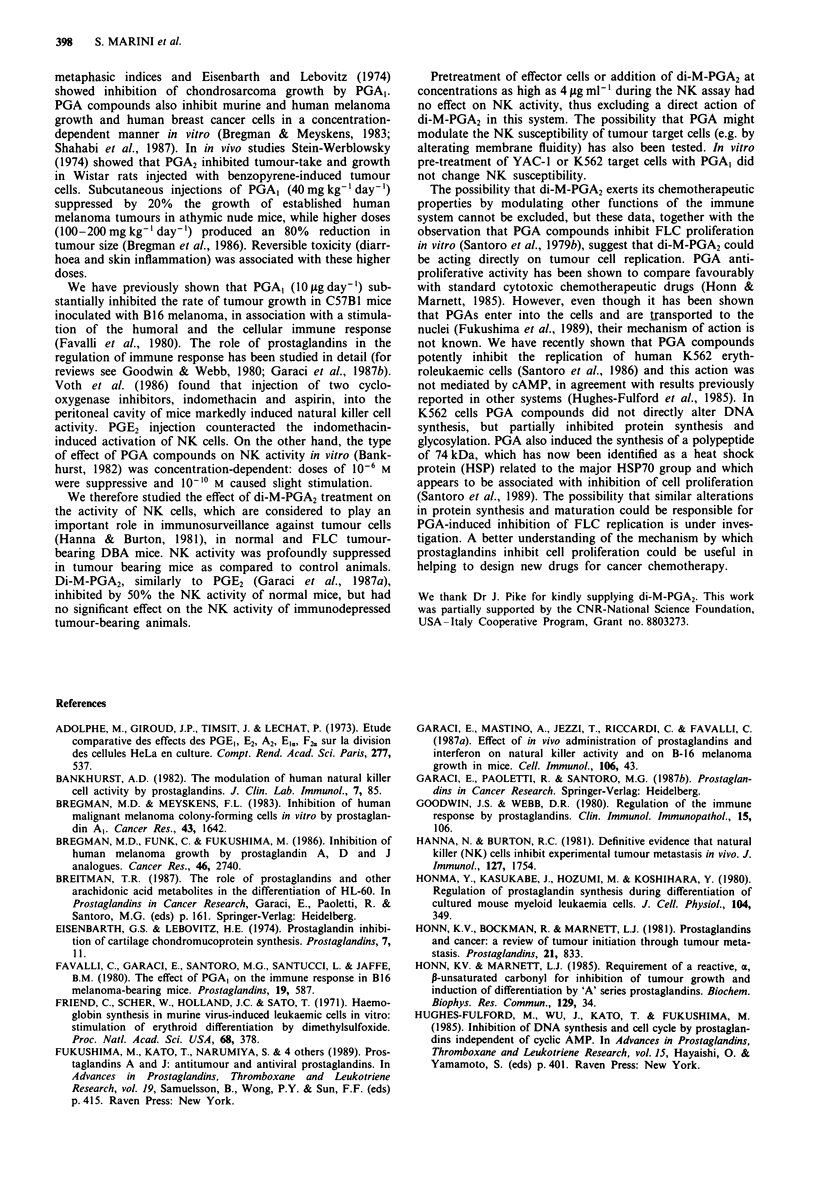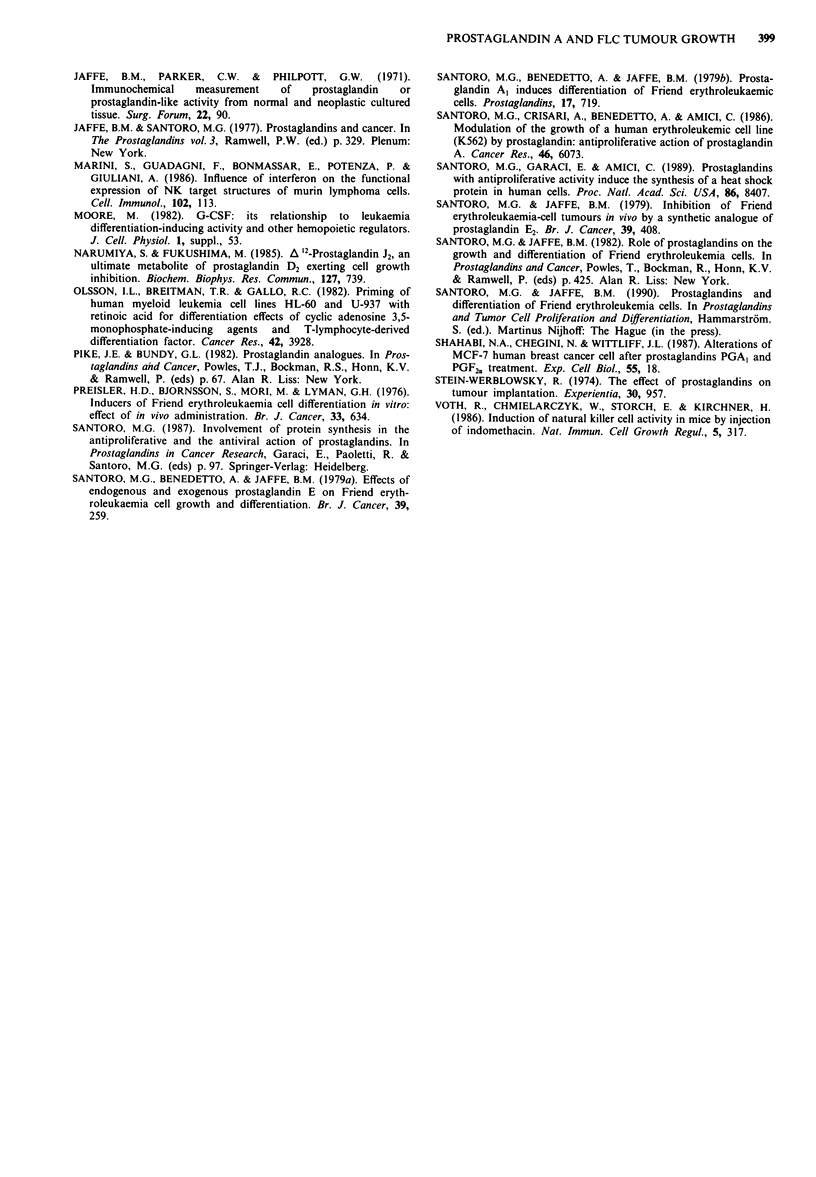# Growth inhibition of Friend erythroleukaemia cell tumours in vivo by a synthetic analogue of prostaglandin A: an action independent of natural killer-activity.

**DOI:** 10.1038/bjc.1990.86

**Published:** 1990-03

**Authors:** S. Marini, A. T. Palamara, E. Garaci, M. G. Santoro

**Affiliations:** Department of Experimental Medicine and Biochemical Sciences, CNR, II University of Rome, Italy.

## Abstract

Prostaglandins of the A series (PGAs) have been previously shown to inhibit the growth and to stimulate the differentiation of Friend erythroleukaemic cells (FLC) in vitro. In the present report we analysed the effect of PGA treatment in vitro on FLC tumorigenicity, and in vivo on FLC proliferation and on natural killer (NK) activity. PGA1 pretreatment of FLC in vitro for 5 days before inoculation into syngeneic mice slightly delayed tumour appearance, but did not significantly alter the pattern of tumour growth or mice survival, indicating that PGA1, at least in the conditions studied, did not affect FLC tumorigenicity. Daily treatment of mice with a long-acting synthetic analogue of PGA2 (16, 16 dimethyl-PGA2-methyl ester, di-M-PGA2) delayed tumour appearance, inhibited tumour growth, as measured by tumour weight and diameter, and increased the median mice survival time by 15-35%, depending on the schedule of treatment. Daily treatment with di-M-PGA2 strongly suppressed NK activity in normal mice but had no significant effect in tumour-bearing immunodepressed mice. PGA treatment of effector or target cells in vitro, or PGA added during the NK assay, had no effect on NK activity. We suggest that the chemotherapeutic effect of PGA is due to a direct action on tumour cell replication rather than to a stimulation of the host NK activity.


Br. J. Cancer (1990), 61, 394-399 © Macmillan Press Ltd., 1990

### Growth inhibition of Friend erythroleukaemia cell tumours *in vivo* by a synthetic analogue of prostaglandin A: an action independent of natural killer-activity

S. Marini[1], A.T. Palamara[1], E. Garaci[1] & M.G. Santoro[1,2]

[1]*Department of Experimental Medicine and Biochemical Sciences and* [2]*Institute of Experimental Medicine, CNR, II University of Rome, Via O. Raimondo, 00173, Rome, Italy.*

**Summary** Prostaglandins of the A series (PGAs) have been previously shown to inhibit the growth and to stimulate the differentiation of Friend erythroleukaemic cells (FLC) *in vitro*. In the present report we analysed the effect of PGA treatment *in vitro* on FLC tumorigenicity, and *in vivo* on FLC proliferation and on natural killer (NK) activity. $PGA_1$ pretreatment of FLC *in vitro* for 5 days before inoculation into syngeneic mice slightly delayed tumour appearance, but did not significantly alter the pattern of tumour growth or mice survival, indicating that $PGA_1$, at least in the conditions studied, did not affect FLC tumorigenicity. Daily treatment of mice with a long-acting synthetic analogue of $PGA_2$ (16, 16 dimethyl-$PGA_2$-methyl ester, di-M-$PGA_2$) delayed tumour appearance, inhibited tumour growth, as measured by tumour weight and diameter, and increased the median mice survival time by 15–35%, depending on the schedule of treatment. Daily treatment with di-M-$PGA_2$ strongly suppressed NK activity in normal mice but had no significant effect in tumour-bearing immunodepressed mice. PGA treatment of effector or target cells *in vitro*, or PGA added during the NK assay, had no effect on NK activity. We suggest that the chemotherapeutic effect of PGA is due to a direct action on tumour cell replication rather than to a stimulation of the host NK activity.

Since the first observation that tumour tissue produces much larger amounts of prostaglandins (PGs) than normal tissue (Jaffe *et al.*, 1971), there has been a great deal of research aimed at evaluating the relationships between PGs and tumour cell growth and functions. PGs are known to be involved in regulating cell proliferation and differentiation in a large number of systems *in vitro* and *in vivo*. However, their action varies with the molecular structure, the dose and the animal model (for reviews see Jaffe & Santoro, 1977; Honn *et al.*, 1981; Garaci *et al.*, 1987*b*). In particular prostaglandin A, E and D compounds (PGAs, PGEs and PGDs respectively) inhibit the growth and/or stimulate the differentiation of several animal and human leukaemic cell lines, among which are WEHI-3B-D$^-$ mouse myelomonocytic leukaemia (Moore, 1982), L-1210 mouse leukaemia (Narumiya & Fukushima, 1985), M1 mouse myeloid leukaemia (Honma *et al.*, 1980), HL-60 human promyelocytic leukaemia (Breitman, 1987), U-937 human lymphoma (Olsson *et al.*, 1982) and K562 human erythroleukaemia (Santoro *et al.*, 1986, 1989; Santoro, 1987). PGs also have been shown to play a role in controlling the growth and differentiation of normal erythroid precursors, and PGE and PGA compounds were found to stimulate erythropoiesis *in vivo* and *in vitro* (for a review see Santoro & Jaffe, 1990).

For several years we have studied the role of prostaglandins in the modulation of growth and differentiation of the virus-induced murine Friend erythroleukaemia cells (FLC). These cells grow indefinitely in suspension culture and can be induced to differentiate *in vitro* from a pro-erythroblast-like to a normoblast-like stage that produces haemoglobin upon stimulation with dimethylsulfoxide (DMSO) and several other agents (Santoro & Jaffe, 1982).

FLC inoculated intravenously (i.v.) into DBA/2 mice produces a malignant disease characterised by leukaemic cell infiltration of marrow, lymph nodes, liver and spleen (Preisler *et al.*, 1976). After subcutaneous (s.c.) inoculation, they produce s.c. tumours similar to myeloblastomas, which cause the death of the animals within 3–7 weeks. Differentiating FLC, i.e. FLC pretreated *in vitro* with DMSO, produce smaller tumours and permit longer mouse survival than do undifferentiated cells (Friend *et al.*, 1971; Preisler *et al.*, 1976).

Correspondence: S. Marini.
Received 30 January 1989; and in revised form 30 October 1989.

We have previously produced evidence that endogenous PGE is involved in regulating FLC differentiation and proliferation *in vitro* (Santoro *et al.*, 1979*a*), and that a $PGE_2$ analogue (16, 16-dimethyl-$PGE_2$-methyl ester; di-M-$PGE_2$) inhibited FLC tumour growth *in vivo* and increased the survival of mice injected s.c. with FLC (Santoro & Jaffe, 1979). We have also shown that PGA compounds are the most effective PGs for inhibiting FLC proliferation *in vitro*, and are the only PGs studied that can stimulate differentiation in the absence of other inducers (Santoro *et al.*, 1979*b*).

The purpose of the present study was to determine whether PGA-treatment could affect FLC tumorigenicity or FLC proliferation *in vivo*. Since PGs can affect the immune response (Goodwin & Webb, 1980), we also examined the effect of PGA on the activation of natural killer cells *in vitro* and *in vivo*. While the natural PGA compounds were used *in vitro*, for *in vivo* studies we used a long-acting synthetic analogue of $PGA_2$, 16, 16-dimethyl-$PGA_2$-methyl ester (di-M-$PGA_2$), which has the same activity as $PGA_1$ on FLC growth and differentiation *in vitro* (Santoro & Jaffe, 1982), and in which the presence of the two methyl groups in position 16 blocks degradation via 15-hydroxy-dehydrogenase (Pike & Bundy, 1982).

### Materials and methods

### *Cell culture*

Friend erythroleukaemia (FLC, strain 745, cell line GM-86 from the Institute for Medical Research, Camden, NJ, USA), K562 erythroleukaemia and YAC-1 thymoma cell lines were grown in RPMI 1640 medium (Flow Laboratories, UK), supplemented with 15% fetal calf serum (FCS) or 10% heat-inactivated fetal bovine serum (FBS) (Flow Laboratories), respectively for FLC and K562 or YAC-1 cells, glutamine 2 mM, penicillin 100 U $ml^{-1}$ and streptomycin 0.1 mg $ml^{-1}$ (Complete Culture Medium, CCM), in a humidified 5% $CO_2$ atmosphere at 37°C. Cell numbers were determined with a haemocytometer; s.e. for 5–10 counts of the same culture varied from 2 to 6%. Cell viability, determined by vital dye exclusion (trypan blue, 0.04%), ranged between 97 and 100%; it was not influenced by $PGA_1$ or ethanol added at the concentrations used. $PGA_1$ (Sigma Chemicals Co., St Louis, MO, USA) and di-M-$PGA_2$, kindly provided by Dr J. Pike, the Upjohn Co. (Kalamazoo, MI, USA), were stored as 100% ethanolic stock solutions (2 mg ml$^{-1}$) at −20°C and diluted in RPMI 1640 (for *in vitro* treatment) or in sterile 154 mM NaCl (for *in vivo* administration) just before use. Control medium contained the same concentration of ethanol diluent (0.01%), which did not affect cell viability, DNA, RNA or protein synthesis (Amici *et al*., unpublished).

*Tumour inoculation*

One hundred and twenty 6-week-old female DBA/2 mice were weighed and injected s.c., under a light ether anaesthesia, in the right flank with 5 × 10$^5$ viable FLC in 0.2 ml RPMI 1640 medium. Mice were randomised into groups of 10–15 animals, and injected intraperitoneally (i.p.) with 100 µl of sterile saline solution containing 10% ethanol (control) or di-M-$PGA_2$ in 10% ethanolic solution. As previously reported (Favalli *et al*., 1980) ethanol diluent was not toxic to the mice and did not significantly affect FLC tumour growth. Tumour appearance was assessed by daily palpation, and when tumours were >2 mm in diameter they were measured daily in at least two dimensions by Vernier caliper; the average of the smallest and the largest diameters was calculated. For two-tailed statistical comparisons Student's *t* test for unpaired data was used and *P* values of <0.05 were considered significant. Curves of rates of tumour appearance and mice survival were compared with the use of the two-tailed signed rank test for paired samples (Epistat computer program) and *P* values of <0.05 were considered significant.

*$^{51}Cr$ labelling of cells and NK assay*

NK activity was detected by using the NK-highly sensitive YAC-1 cell line. YAC-1 thymoma was maintained in CCM. Labelling of cells and measurement of NK activity were performed as previously described (Marini *et al*., 1985). For di-M-$PGA_2$ treatment of target cells, YAC-1 cells were suspended in 8 ml of CCM (10$^6$ cells ml$^{-1}$) and then incubated overnight at 37°C in the presence of 2 µg ml$^{-1}$ di-M-$PGA_2$. Tumour cells were then washed three times with phosphate buffer solution, pH 7.2 (PBS), counted, resuspended in CCM and used in assays. In other experiments di-M-$PGA_2$ (2 µg ml$^{-1}$) was added to the medium during the test. As described in the text, in one experiment K562 human erythroleukaemic cells were used as targets and peripheral blood lymphocytes from a healthy human donor were used as attacker cells.

To prepare labelled target cells for cytotoxicity assays, YAC-1 cells were removed from continuous culture, centrifuged (250 *g* for 10 min) and resuspended in 0.1 ml of FBS. After addition of 100 µCi sodium chromate ($^{51}Cr$) (Amersham International, Amersham, UK), the cells were incubated for 1 h at 37°C in a 5% $CO_2$ humidified atmosphere. Labelled cells were washed three times with RPMI 1640 containing 10 mM HEPES, 2% heat-inactivated FBS and 50 µg ml$^{-1}$ gentamicin, and then resuspended in another aliquot of the same medium supplemented with 10% FBS (Chromium Release Assay medium, CRA). The viable cell number was then adjusted to 10$^5$ ml$^{-1}$. Assays were performed in U-shaped 96-well microtitre plates (Greiner C.A. and Sohne, Nurtingen, FRG). After serial two-fold dilution starting at 10$^7$ cells ml$^{-1}$, effector spleen cells were plated in 0.1 ml CRA and labelled target cells in 0.1 ml were added. Plates were then incubated for 4 h at 37°C in a 5% $CO_2$ incubator, centrifuged (800 *g* for 10 min) and radioactivity in 0.1 ml of supernatant was measured in a Packard γ scintillation counter. All assays were performed in quadruplicate and four effector: target (E:T) ratios were employed. The baseline $^{51}Cr$ release was determined in microwells in which splenocytes were replaced by unlabelled target cells (i.e. autologous control, AC). The AC never exceeded 10% of the total radioactivity incorporated by target cells. Results are expressed as the percentage of specific lysis and calculated as:

$$\% \text{ specific lysis} = \frac{\text{c.p.m. TS} - \text{c.p.m AC}}{\text{c.p.m. TC}} \times 100$$

where c.p.m. TS is the mean value of the counts per minute (c.p.m.) of the test sample in the presence of effector cells; c.p.m. AC is the mean c.p.m. of the AC, and c.p.m. TC is the mean c.p.m. corresponding to the total amount of $^{51}Cr$ incorporated into target cells.

### Results

*PGA effect on FLC tumorigenicity*

Friend erythroleukaemic cells derived from a logarithmically growing population, were plated at a density of 2.5 × 10$^5$ cells ml$^{-1}$ in CCM, and $PGA_1$ (2 µg ml$^{-1}$) or ethanol diluent were added. Treatment with $PGA_1$ was not repeated during the experiment. As it has been previously reported (Santoro *et al*., 1979*b*) $PGA_1$ only partially inhibited the proliferation of FLC derived from a logarithmically growing population. In fact, if treatment was not repeated after 48 h, in contrast to FLC derived from stationary-phase cultures, these cells regained their growth potential and no significant inhibition of cell proliferation was found after 96 h of $PGA_1$ treatment (data not shown). After 5 days the cells were counted, washed twice in PBS, resuspended in RPMI 1640 devoid of FCS, and inoculated s.c. into DBA/2 mice (5 × 10$^5$ cells per mouse) as described in Materials and methods. The rate of tumour appearance, the pattern of tumour growth and mouse survival rate were compared with those of mice injected with untreated FLC. Eight days after tumour inoculation, 46% of the control animals showed a visible tumour, compared with 6% of the mice injected with pretreated FLC ($P<0.01$).

This initial difference decreased in the following days and almost completely disappeared 14 to 16 days after inoculation (Figure 1a). $PGA_1$-pretreatment of FLC had no significant effect on mouse survival (Figure 1b) or on tumour growth (at day 16 post-injection the tumour diameters were: 18.4 ± 3.5 mm in controls and 14.9 ± 3.9 mm in $PGA_1$-pretreated mice, $P<0.1$).

*Effect of di-M-$PGA_2$ treatment in vivo*

In two separate experiments untreated, undifferentiated FLC derived from a logarithmically growing population were injected in DBA/2 mice as previously described. After 2–3 h the mice were randomised (10–15 mice per group) and injected i.p. with 100 µl of sterile 154 mM NaCl containing 10 µg di-M-$PGA_2$. Injections were repeated daily up to day 21 p.i. (protocol A), or to day 16 (protocol B). Control mice were treated with the same amount of diluent. Tumour appearance was slightly delayed by di-M-$PGA_2$ treatment (6 days p.i. visible tumours were present in 75% of PG-treated as compared to 92% of control mice in protocol A, and 80% as compared to 100% of control in protocol B; $P<0.01$). After appearance, the tumours grew rapidly in a rather uniform spherical shape, and their diameters were measured daily (Figure 2a, b). In protocol B at day 16 p.i. the mice were killed and tumours were removed and weighed. Results are illustrated in Table I. Di-M-$PGA_2$ treatment reduced the rate of tumour growth measured both as tumour diameter ($P<0.05$) and tumour weight ($P<0.02$). PG-treatment, even though it caused diarrhoea in the first hour after injection, did not alter the weight of tumour-bearing (Table I) or normal DBA/2 mice (after 15 days of treatment the weights were: 16.1 ± 0.85 g in the controls and 16.73 ± 0.09 g in the di-M-$PGA_2$-treated mice).

Di-M-$PGA_2$ treatment increased mouse survival. Figure 3 shows the survival curves of animals that had been injected with di-M-$PGA_2$ (10 µg per injection) once daily for 21 days (group I, Figure 3a), or twice daily for 13 days, followed by

**Figure 1** Effect of $PGA_1$ treatment *in vitro* on the tumorigenicity of undifferentiated FLC. FLC treated for 5 days with $PGA_1$ (2 μg $ml^{-1}$, only once after plating, ●——●), or control diluent (○——○) were inoculated into two groups of 14 mice each. **a**, The rate of tumour appearance. In mice that received $PGA_1$-treated FLC, the appearance of tumours was delayed ($P<0.01$). **b**, Mouse survival. No difference in per cent survival was observed between the two groups, up to 100 days post-injection.

**Figure 2** Effect of di-M-$PGA_2$ treatment *in vivo* on FLC tumour growth. **a**, 24 mice were inoculated with $5 \times 10^5$ FLC and divided into two groups which received either di-M-$PGA_2$ (10 μg per day per mouse, $n = 12$, □——□) or control diluent ($n = 12$, ■——■) up to 21 days post-injection. Data are expressed as mean tumour diameter ± s.d. ($P<0.05$ on days 10, 14). **b**, After FLC inoculation ($5 \times 10^5$ cells per mouse) 20 mice were divided into two groups and treated with di-M-$PGA_2$ (10 μg per day per mouse, □——□, $n = 10$) or control diluent (■——■, $n = 10$) for 16 days. Tumours from di-M-$PGA_2$-treated mice were smaller than controls ($P<0.05$ on days 14, 16).

**Table I** Effect of di-M-$PGA_2$ treatment on FLC tumour growth

| | *Control* | *Di-M-$PGA_2$* | *% inhibition* |
|---|---|---|---|
| Tumour diameter (mm) | 21.40 ± 1.18 | 15.02 ± 2.02* | 30 |
| (mean ± s.d.) | ($n = 11$) | ($n = 11$) | |
| Tumour weight (g) | 2.03 ± 0.51 | 1.14 ± 0.33** | 44 |
| (mean ± s.d.) | ($n = 5$) | ($n = 5$) | |
| Total body weight (g) | 17.28 ± 0.32 | 16.87 ± 0.82 | – |
| (mean ± s.d.) | ($n = 18$) | ($n = 18$) | |

Measurements of tumour diameters and weights were determined on day 16 after tumour inoculation. *$P<0.05$; **$P<0.02$.

one injection per day for a further 10 days (group II, Figure 3b). Median survival was increased by 15% in group I ($P<0.01$) and by 35% in group II ($P<0.005$). However, while it significantly increased mice median survival, treatment with the twice daily dose of di-M-$PGA_2$ had no effect on tumour appearance (day 6 p.i.: controls = 54%; di-M-$PGA_2$ = 46%; $P<0.5$), possibly due to an effect on the immune system in the first days after tumour inoculation, when the animals are not yet immunodepressed by tumour growth.

### *Measurement of NK activity in normal and tumour-bearing mice after di-M-$PGA_2$ treatment*

In order to evaluate whether the anti-tumour effect of di-M-$PGA_2$ is mediated by a modulation of NK activity, 36 five-week-old mice were inoculated s.c. with $5 \times 10^5$ Friend erythroleukaemic cells, in two separate experiments, and treated with di-M-$PGA_2$ (10 μg per day per mouse) or control diluent for the following 15 days; 14 animals not inoculated with FLC were randomly divided into two groups and treated identically. Spleens from control and tumour-bearing animals were collected after 15 days of treatment. Figure 4 shows that at 15 days p.i. the NK activity was 45% less in animals inoculated with FLC compared to normal controls ($P<0.02$). Di-M-$PGA_2$ treatment strongly suppressed (52%) NK activity in normal mice but it did not further decrease the depressed NK activity in tumour-bearing mice (Figure 4b).

In order to investigate whether the immune-modulating action of di-M-$PGA_2$ could be mimicked *in vitro*, the effect of di-M-$PGA_2$ was studied on NK activity either by separately treating effector and target cells or by adding di-M-$PGA_2$ directly during NK assay. Spleen cells ($10^6$ $ml^{-1}$ in 10 ml

**Figure 3** Effect of di-M-$PGA_2$ treatment *in vivo* on survival of mice injected with undifferentiated FLC. **a**, Di-M-$PGA_2$ treatment (10 µg per day per mouse, $n = 12$), was started on the day of tumour inoculation ($5 \times 10^5$ cells per mouse) and continued for 21 days. Mean survival was higher in di-M-$PGA_2$-treated (Δ——Δ) as compared to control (▲——▲) mice ($P<0.01$). **b**, Di-M-$PGA_2$ treatment (10 µg per mouse) was started on the day of tumour inoculation and repeated twice daily for the first 13 days and once daily for a further 10 days. Control mice received ethanol diluent following the same schedule. Per cent survival was increased in di-M-$PGA_2$-treated mice (Δ——Δ, $n = 11$) compared to controls (▲——▲, $n = 11$) ($P<0.005$).

**Figure 4** Effect of di-M-$PGA_2$ treatment *in vivo* on NK activity of normal or tumour-bearing animals. Normal or FLC-inoculated mice were treated with di-M-$PGA_2$ (10 µg per mouse per day) or ethanol diluent for 15 days after tumour inoculation. The last day of treatment mice were killed, the spleens were collected and NK activity was measured in both normal (**a**) and tumour-bearing (**b**) animals either untreated (O——O) or treated with di-M-$PGA_2$ (●——●). Four mice were tested for each group and each mouse was tested individually in quadruplicate. NK activity was lower in di-M-$PGA_2$-treated normal mice ($P<0.02$) but the analogue had little or no effect in immunodepressed tumour-bearing animals.

CCM) from normal animals were treated *in vitro* for 4 h with 2 µg $ml^{-1}$ di-M-$PGA_2$ or control diluent and thereafter used as attacker cells in the NK assay. This short-term *in vitro* di-M-$PGA_2$ treatment did not modulate NK activity (25.4 ± 0.9% cytotoxicity in controls versus 24.0 ± 0.3% cytotoxicity in PG-treated spleen cells, E:T ratio 100:1). NK activity of splenocytes tested after 20 h of incubation with di-M-$PGA_2$ or control solutions was too low to be measured.

Further experiments have been performed which demonstrate that di-M-$PGA_2$ activity was not due to a modulation of NK susceptibility in target tumour cells. YAC-1 cells or the human erythroleukaemia K562 cells ($10^6$ cells $ml^{-1}$) were treated overnight with di-M-$PGA_2$ (2 µg $ml^{-1}$) and used as targets for NK assay, after $^{51}Cr$-labelling. Di-M-$PGA_2$-treated target cells showed similar NK susceptibility compared to control cells (for YAC-1 target cells: controls 34.5 ± 1.7%, di-M-$PGA_2$ 34.8 ± 1.4% cytotoxicity; for K562 target cells: controls 77.7 ± 0.8%, di-M-$PGA_2$ 75.6 ± 1.0% cytotoxicity; E:T ratio 100:1).

Finally, addition of di-M-$PGA_2$ (2 µg $ml^{-1}$) or ethanol control during the NK test (with YAC-1 cells) did not cause any change in the cytotoxicity (controls 26.2 ± 0.9%; ethanol controls 27.8 ± 1.3%; di-M-$PGA_2$ 29.1 ± 1.2% cytotoxicity; E:T ratio 100:1).

### Discussion

The results described in this paper show that systemic administration of a long-acting analogue of $PGA_2$, di-M-$PGA_2$, inhibited FLC tumour growth *in vivo*. All the variables measured, i.e. tumour time of appearance, diameter and weight, were reduced by PG-treatment while the length of survival of PG-treated mice was increased. These effects were similar to those obtained with di-M-$PGE_2$ *in vivo* (Santoro & Jaffe, 1979); they were dose-dependent and occurred at doses that did not produce weight loss in the mice. On the other hand, when FLC treated *in vitro* with $PGA_1$ (2 µg $ml^{-1}$) were injected into DBA mice, even though the time of tumour onset was slightly delayed, no difference in the rate of tumour growth or in mice survival was found.

The anti-tumour activity of PGA compounds has been previously reported in different animal models *in vitro* and *in vivo*. In 1973 Adolphe *et al.* reported that $PGA_2$ inhibited HeLa cell proliferation as measured by mitotic and metaphasic indices and Eisenbarth and Lebovitz (1974) showed inhibition of chondrosarcoma growth by $PGA_1$. PGA compounds also inhibit murine and human melanoma growth and human breast cancer cells in a concentration-dependent manner *in vitro* (Bregman & Meyskens, 1983; Shahabi *et al.*, 1987). In *in vivo* studies Stein-Werblowsky (1974) showed that $PGA_2$ inhibited tumour-take and growth in Wistar rats injected with benzopyrene-induced tumour cells. Subcutaneous injections of $PGA_1$ (40 mg $kg^{-1}$ $day^{-1}$) suppressed by 20% the growth of established human melanoma tumours in athymic nude mice, while higher doses (100–200 mg $kg^{-1}$ $day^{-1}$) produced an 80% reduction in tumour size (Bregman *et al.*, 1986). Reversible toxicity (diarrhoea and skin inflammation) was associated with these higher doses.

We have previously shown that $PGA_1$ (10 $\mu$g $day^{-1}$) substantially inhibited the rate of tumour growth in C57B1 mice inoculated with B16 melanoma, in association with a stimulation of the humoral and the cellular immune response (Favalli *et al.*, 1980). The role of prostaglandins in the regulation of immune response has been studied in detail (for reviews see Goodwin & Webb, 1980; Garaci *et al.*, 1987*b*). Voth *et al.* (1986) found that injection of two cyclo-oxygenase inhibitors, indomethacin and aspirin, into the peritoneal cavity of mice markedly induced natural killer cell activity. $PGE_2$ injection counteracted the indomethacin-induced activation of NK cells. On the other hand, the type of effect of PGA compounds on NK activity *in vitro* (Bankhurst, 1982) was concentration-dependent: doses of $10^{-6}$ M were suppressive and $10^{-10}$ M caused slight stimulation.

We therefore studied the effect of di-M-$PGA_2$ treatment on the activity of NK cells, which are considered to play an important role in immunosurveillance against tumour cells (Hanna & Burton, 1981), in normal and FLC tumour-bearing DBA mice. NK activity was profoundly suppressed in tumour bearing mice as compared to control animals. Di-M-$PGA_2$, similarly to $PGE_2$ (Garaci *et al.*, 1987*a*), inhibited by 50% the NK activity of normal mice, but had no significant effect on the NK activity of immunodepressed tumour-bearing animals.

Pretreatment of effector cells or addition of di-M-$PGA_2$ at concentrations as high as 4 $\mu$g $ml^{-1}$ during the NK assay had no effect on NK activity, thus excluding a direct action of di-M-$PGA_2$ in this system. The possibility that PGA might modulate the NK susceptibility of tumour target cells (e.g. by alterating membrane fluidity) has also been tested. *In vitro* pre-treatment of YAC-1 or K562 target cells with $PGA_1$ did not change NK susceptibility.

The possibility that di-M-$PGA_2$ exerts its chemotherapeutic properties by modulating other functions of the immune system cannot be excluded, but these data, together with the observation that PGA compounds inhibit FLC proliferation *in vitro* (Santoro *et al.*, 1979*b*), suggest that di-M-$PGA_2$ could be acting directly on tumour cell replication. PGA anti-proliferative activity has been shown to compare favourably with standard cytotoxic chemotherapeutic drugs (Honn & Marnett, 1985). However, even though it has been shown that PGAs enter into the cells and are transported to the nuclei (Fukushima *et al.*, 1989), their mechanism of action is not known. We have recently shown that PGA compounds potently inhibit the replication of human K562 erythroleukaemic cells (Santoro *et al.*, 1986) and this action was not mediated by cAMP, in agreement with results previously reported in other systems (Hughes-Fulford *et al.*, 1985). In K562 cells PGA compounds did not directly alter DNA synthesis, but partially inhibited protein synthesis and glycosylation. PGA also induced the synthesis of a polypeptide of 74 kDa, which has now been identified as a heat shock protein (HSP) related to the major HSP70 group and which appears to be associated with inhibition of cell proliferation (Santoro *et al.*, 1989). The possibility that similar alterations in protein synthesis and maturation could be responsible for PGA-induced inhibition of FLC replication is under investigation. A better understanding of the mechanism by which prostaglandins inhibit cell proliferation could be useful in helping to design new drugs for cancer chemotherapy.

We thank Dr J. Pike for kindly supplying di-M-$PGA_2$. This work was partially supported by the CNR-National Science Foundation, USA–Italy Cooperative Program, Grant no. 8803273.

### References

ADOLPHE, M., GIROUD, J.P., TIMSIT, J. & LECHAT, P. (1973). Etude comparative des effects des $PGE_1$, $E_2$, $A_2$, $E_{1\alpha}$, $F_{2\alpha}$ sur la division des cellules HeLa en culture. *Compt. Rend. Acad. Sci. Paris*, **277**, 537.

BANKHURST, A.D. (1982). The modulation of human natural killer cell activity by prostaglandins. *J. Clin. Lab. Immunol.*, **7**, 85.

BREGMAN, M.D. & MEYSKENS, F.L. (1983). Inhibition of human malignant melanoma colony-forming cells *in vitro* by prostaglandin $A_1$. *Cancer Res.*, **43**, 1642.

BREGMAN, M.D., FUNK, C. & FUKUSHIMA, M. (1986). Inhibition of human melanoma growth by prostaglandin A, D and J analogues. *Cancer Res.*, **46**, 2740.

BREITMAN, T.R. (1987). The role of prostaglandins and other arachidonic acid metabolites in the differentiation of HL-60. In *Prostaglandins in Cancer Research*, Garaci, E., Paoletti, R. & Santoro, M.G. (eds) p. 161. Springer-Verlag: Heidelberg.

EISENBARTH, G.S. & LEBOVITZ, H.E. (1974). Prostaglandin inhibition of cartilage chondromucoprotein synthesis. *Prostaglandins*, **7**, 11.

FAVALLI, C., GARACI, E., SANTORO, M.G., SANTUCCI, L. & JAFFE, B.M. (1980). The effect of $PGA_1$ on the immune response in B16 melanoma-bearing mice. *Prostaglandins*, **19**, 587.

FRIEND, C., SCHER, W., HOLLAND, J.C. & SATO, T. (1971). Haemoglobin synthesis in murine virus-induced leukaemic cells in vitro: stimulation of erythroid differentiation by dimethylsulfoxide. *Proc. Natl. Acad. Sci. USA*, **68**, 378.

FUKUSHIMA, M., KATO, T., NARUMIYA, S. & 4 others (1989). Prostaglandins A and J: antitumour and antiviral prostaglandins. In *Advances in Prostaglandins, Thromboxane and Leukotriene Research, vol. 19*, Samuelsson, B., Wong, P.Y. & Sun, F.F. (eds) p. 415. Raven Press: New York.

GARACI, E., MASTINO, A., JEZZI, T., RICCARDI, C. & FAVALLI, C. (1987*a*). Effect of *in vivo* administration of prostaglandins and interferon on natural killer activity and on B-16 melanoma growth in mice. *Cell. Immunol.*, **106**, 43.

GARACI, E., PAOLETTI, R. & SANTORO, M.G. (1987*b*). *Prostaglandins in Cancer Research*. Springer-Verlag: Heidelberg.

GOODWIN, J.S. & WEBB, D.R. (1980). Regulation of the immune response by prostaglandins. *Clin. Immunol. Immunopathol.*, **15**, 106.

HANNA, N. & BURTON, R.C. (1981). Definitive evidence that natural killer (NK) cells inhibit experimental tumour metastasis *in vivo*. *J. Immunol.*, **127**, 1754.

HONMA, Y., KASUKABE, J., HOZUMI, M. & KOSHIHARA, Y. (1980). Regulation of prostaglandin synthesis during differentiation of cultured mouse myeloid leukaemia cells. *J. Cell. Physiol.*, **104**, 349.

HONN, K.V., BOCKMAN, R. & MARNETT, L.J. (1981). Prostaglandins and cancer: a review of tumour initiation through tumour metastasis. *Prostaglandins*, **21**, 833.

HONN, KV. & MARNETT, L.J. (1985). Requirement of a reactive, $\alpha$, $\beta$-unsaturated carbonyl for inhibition of tumour growth and induction of differentiation by 'A' series prostaglandins. *Biochem. Biophys. Res. Commun.*, **129**, 34.

HUGHES-FULFORD, M., WU, J., KATO, T. & FUKUSHIMA, M. (1985). Inhibition of DNA synthesis and cell cycle by prostaglandins independent of cyclic AMP. In *Advances in Prostaglandins, Thromboxane and Leukotriene Research, vol. 15*, Hayaishi, O. & Yamamoto, S. (eds) p. 401. Raven Press: New York.

JAFFE, B.M., PARKER, C.W. & PHILPOTT, G.W. (1971). Immunochemical measurement of prostaglandin or prostaglandin-like activity from normal and neoplastic cultured tissue. *Surg. Forum*, **22**, 90.

JAFFE, B.M. & SANTORO, M.G. (1977). Prostaglandins and cancer. In *The Prostaglandins vol. 3*, Ramwell, P.W. (ed.) p. 329. Plenum: New York.

MARINI, S., GUADAGNI, F., BONMASSAR, E., POTENZA, P. & GIULIANI, A. (1986). Influence of interferon on the functional expression of NK target structures of murin lymphoma cells. *Cell. Immunol.*, **102**, 113.

MOORE, M. (1982). G-CSF: its relationship to leukaemia differentiation-inducing activity and other hemopoietic regulators. *J. Cell. Physiol.* **1**, suppl., 53.

NARUMIYA, S. & FUKUSHIMA, M. (1985). $\Delta^{12}$-Prostaglandin $J_2$, an ultimate metabolite of prostaglandin $D_2$ exerting cell growth inhibition. *Biochem. Biophys. Res. Commun.*, **127**, 739.

OLSSON, I.L., BREITMAN, T.R. & GALLO, R.C. (1982). Priming of human myeloid leukemia cell lines HL-60 and U-937 with retinoic acid for differentiation effects of cyclic adenosine 3,5-monophosphate-inducing agents and T-lymphocyte-derived differentiation factor. *Cancer Res.*, **42**, 3928.

PIKE, J.E. & BUNDY, G.L. (1982). Prostaglandin analogues. In *Prostaglandins and Cancer*, Powles, T.J., Bockman, R.S., Honn, K.V. & Ramwell, P. (eds) p. 67. Alan R. Liss: New York.

PREISLER, H.D., BJORNSSON, S., MORI, M. & LYMAN, G.H. (1976). Inducers of Friend erythroleukaemia cell differentiation *in vitro*: effect of *in vivo* administration. *Br. J. Cancer*, **33**, 634.

SANTORO, M.G. (1987). Involvement of protein synthesis in the antiproliferative and the antiviral action of prostaglandins. In *Prostaglandins in Cancer Research*, Garaci, E., Paoletti, R. & Santoro, M.G. (eds) p. 97. Springer-Verlag: Heidelberg.

SANTORO, M.G., BENEDETTO, A. & JAFFE, B.M. (1979*a*). Effects of endogenous and exogenous prostaglandin E on Friend erythroleukaemia cell growth and differentiation. *Br. J. Cancer*, **39**, 259.

SANTORO, M.G., BENEDETTO, A. & JAFFE, B.M. (1979*b*). Prostaglandin $A_1$ induces differentiation of Friend erythroleukaemic cells. *Prostaglandins*, **17**, 719.

SANTORO, M.G., CRISARI, A., BENEDETTO, A. & AMICI, C. (1986). Modulation of the growth of a human erythroleukemic cell line (K562) by prostaglandin: antiproliferative action of prostaglandin A. *Cancer Res.*, **46**, 6073.

SANTORO, M.G., GARACI, E. & AMICI, C. (1989). Prostaglandins with antiproliferative activity induce the synthesis of a heat shock protein in human cells. *Proc. Natl. Acad. Sci. USA*, **86**, 8407.

SANTORO, M.G. & JAFFE, B.M. (1979). Inhibition of Friend erythroleukaemia-cell tumours *in vivo* by a synthetic analogue of prostaglandin $E_2$. *Br. J. Cancer*, **39**, 408.

SANTORO, M.G. & JAFFE, B.M. (1982). Role of prostaglandins on the growth and differentiation of Friend erythroleukemia cells. In *Prostaglandins and Cancer*, Powles, T., Bockman, R., Honn, K.V. & Ramwell, P. (eds) p. 425. Alan R. Liss: New York.

SANTORO, M.G. & JAFFE, B.M. (1990). Prostaglandins and differentiation of Friend erythroleukemia cells. In *Prostaglandins and Tumor Cell Proliferation and Differentiation*, Hammarström. S. (ed.). Martinus Nijhoff: The Hague (in the press).

SHAHABI, N.A., CHEGINI, N. & WITTLIFF, J.L. (1987). Alterations of MCF-7 human breast cancer cell after prostaglandins $PGA_1$ and $PGF_{2\alpha}$ treatment. *Exp. Cell Biol.*, **55**, 18.

STEIN-WERBLOWSKY, R. (1974). The effect of prostaglandins on tumour implantation. *Experientia*, **30**, 957.

VOTH, R., CHMIELARCZYK, W., STORCH, E. & KIRCHNER, H. (1986). Induction of natural killer cell activity in mice by injection of indomethacin. *Nat. Immun. Cell Growth Regul.*, **5**, 317.